# Our New Year’s Greeting

**Published:** 1885-01

**Authors:** 


### HALL'S JOURNAL OF HEALTH

VOL. 32. JANUARY, 1885. NO. 1.

### OUR NEW YEAR'S GREETING.

In this, the opening number of our thirty-second year, our readers will notice some important and attractive changes in the appearance of the JOURNAL OF HEALTH.

The managers of this publication have hitherto held extremely conservative views with regard to changing in any way the appearance of a magazine whose reputation was won by the character of its teachings and the sound good-sense of its literary productions, rather than by the attractiveness of its exterior.

In these days, however, when the art of book-making has been carried to a degree of perfection which makes a printed page "a thing of beauty," it seems consistent that the mechanical work necessary for the publication of the JOURNAL should be done in such a manner as to make the magazine in style and attractiveness worthy of its contents, as well as a rival in beauty of the host of contemporaneous publications.

The designing of our cover page and the selection of the paper on which the JOURNAL is printed was entrusted to a New York decorative artist of well-known ability, and we have no fear that our readers will have cause to criticise his good taste.

With respect to the character of the literary productions which will appear in our pages during the year, we would say that they will consist of original articles by the Editor and physicians of known standing, together with extracts from American and foreign publications, bearing on the great subject of *Health and how to keep it.*

Occasional articles upon other popular subjects will be added, but the fact that this is a Health Journal will render them of secondary importance, and they will only be used to enliven our pages, as otherwise they might appear too technical to be interesting to the reader who desires a popular magazine.

We desire, in closing, to apologize for the delay in issuing this number, which was unavoidable because of the time required for making the changes and improvements spoken of above; but our friends may hereafter rely on receiving the JOURNAL on the 20th of the month preceding that for which it is dated, as we intend to make a point of promptness in publication.

